# Impact of Blinatumomab Treatment on Bone Marrow Function in Patients with Relapsed/Refractory B-Cell Precursor Acute Lymphoblastic Leukemia

**DOI:** 10.3390/cancers13225607

**Published:** 2021-11-09

**Authors:** Hagop M. Kantarjian, Gerhard Zugmaier, Monika Brüggemann, Brent L. Wood, Heinz A. Horst, Yi Zeng, Giovanni Martinelli

**Affiliations:** 1 Department of Leukemia, The University of Texas MD Anderson Cancer Center, Houston, TX 77030, USA; hkantarjian@mdanderson.org; 2 Amgen Research (Munich) GmbH, Staffelseestraße 2, 81477 Munich, Germany; 3 Department of Hematology, University Hospital of Schleswig-Holstein, 24105 Kiel, Germany; m.brueggemann@med2.uni-kiel.de; 4 Department of Hematopathology, Children’s Hospital Los Angeles, Keck School of Medicine, University of Southern California, Los Angeles, CA 90007, USA; bwood@chla.usc.edu; 5 Department of Internal Medicine II, University Hospital of Schleswig-Holstein, 24105 Kiel, Germany; h.horst@med2.uni-kiel.de; 6 Amgen Inc., Thousand Oaks, CA 91320, USA; yzeng01@amgen.com; 7 IRCCS Istituto Romagnolo per lo Studio dei Tumori (IRST) “Dino Amadori”, 47014 Meldola, Italy; giovanni.martinelli@irst.emr.it

**Keywords:** acute lymphoblastic leukemia, bispecific antibody, bispecific T-cell engager, blinatumomab, measurable residual disease, myelosuppression

## Abstract

**Simple Summary:**

Myelosuppression is a side effect of chemotherapy, in which the production of red and white blood cells and platelets is reduced, increasing risk of infections. Blinatumomab, a bispecific T-cell engager (BiTE^®^) molecule, is a novel anticancer drug that kills acute lymphoblastic leukemia (ALL) cells while sparing majority of the normal bone marrow cells. Results from our study, which compared the effects of blinatumomab treatment with chemotherapy on bone marrow function in a large number of patients with ALL, indicated that the decrease in blood cell counts was more severe and lasted longer after chemotherapy compared with blinatumomab treatment, in which the effects were transient. Survival in patients treated with blinatumomab achieving complete remission was more similar between those with incomplete recovery of blood cell counts versus those with complete blood cell counts than the corresponding survival outcomes seen with chemotherapy. In conclusion, blinatumomab treatment caused transient myelosuppression when compared with chemotherapy.

**Abstract:**

Association of blinatumomab treatment with myelosuppression was examined in this study. Peripheral blood counts were assessed prior to, during, and after blinatumomab treatment in patients with relapsed/refractory Philadelphia chromosome-negative (Ph−) B-cell precursor (BCP) acute lymphoblastic leukemia (ALL; *n* = 267) and Ph+ BCP-ALL (*n* = 45) from the TOWER and ALCANTARA studies, respectively, or chemotherapy in patients with Ph− BCP-ALL (*n* = 109) from the TOWER study; all the patients with relapsed/refractory BCP-ALL and responders achieving complete remission (CR) or CR with partial/incomplete hematological recovery (CRh/CRi) were evaluated. Event-free survival (EFS) and overall survival (OS) were assessed in patients achieving CR and CRh/CRi. Median leukocyte, neutrophil, and platelet counts increased during two blinatumomab cycles but remained low longer after chemotherapy. Among the responders, there was a trend that a greater proportion of patients achieved CR with blinatumomab (Ph−, 76.5%; Ph+, 77.8%) versus with chemotherapy (Ph−, 63.6%). In the TOWER study, the survival prognosis for patients achieving CRh/CRi versus CR with blinatumomab was more similar (median OS, 11.9 (95% CI, 3.9–not estimable (NE)) vs. 15.0 (95% CI, 10.4–NE) months, *p* = 0.062) than with chemotherapy (5.2 (95% CI, 1.6–NE) vs. 18.9 (95% CI, 9.3–NE) months, *p* = 0.013). Blinatumomab treatment, with only temporary and transient myelosuppression, resulted in a greater survival benefit than chemotherapy.

## 1. Introduction

Myelosuppression, a common side effect of cytotoxic chemotherapy, can lead to severe persistent leukopenia, neutropenia, and thrombocytopenia in patients with acute lymphoblastic leukemia (ALL) [1,2,3,4,5]. Severe leukopenia and neutropenia are associated with potentially life-threatening infections [6,7,8,9,10,11]. High rates of grade ≥ 3 neutropenia / febrile neutropenia (58–64%) and infections (52–65%) are observed in children, adolescents, and adults with ALL treated with cytotoxic chemotherapy, underlying the high incidence of myelosuppression-related deaths [12,13,14]. In adult patients with ALL treated with cytotoxic chemotherapy, complete remission (CR) with partial hematologic recovery (CRh) and CR with incomplete hematologic recovery (CRi) are associated with worse overall survival (OS) when compared with CR [15,16]. Myelosuppression is also associated with an increased risk of infection by severe acute respiratory syndrome coronavirus 2 (SARS-CoV-2) and death due to COVID-19 [17,18,19]. Thus, for the treatment of patients with ALL, the clinical benefit of cytotoxic chemotherapy must be weighed against these risks due to myelosuppression.

Blinatumomab is a bispecific T-cell engager (BiTE^®^) molecule that activates CD3+ T cells to lyse CD19+ leukemia cells [20,21]. Blinatumomab has demonstrated improved efficacy versus chemotherapy among adults and children with relapsed/refractory (R/R) B-cell precursor (BCP) ALL and induced high rates of MRD remission in adults and children with R/R BCP ALL [12,13,22,23,24,25]. Results from a previously conducted single-arm phase 2 study in a small cohort of 36 adults with R/R BCP ALL demonstrated that blinatumomab treatment was associated with transient and reversible neutropenia and thrombocytopenia in patients who responded to treatment [5]. Herein, we examined a large cohort of patients with Philadelphia chromosome-negative (Ph−) and Philadelphia chromosome-positive (Ph+) R/R BCP ALL from a phase 3 (TOWER) study and a phase 2 (ALCANTARA) study, respectively, to determine if treatment with blinatumomab was associated with myelosuppression. In addition, impact of blinatumomab on bone marrow function is also compared with that of chemotherapy treatment in patients from the TOWER study. Patients with both Ph− and Ph+ R/R BCP ALL were included given that the mechanism of action and efficacy of blinatumomab are independent of high-risk genetic abnormalities [23,26].

## 2. Materials and Methods

### 2.1. Trial Design, Oversight, and Participants

In the randomized, controlled, multicenter phase 3 TOWER trial (NCT02013167), adults (≥ 18 years old) with Ph− R/R BCP ALL were enrolled. Of all the patients randomized to receive blinatumomab (*n* = 271) or chemotherapy (*n* = 134) in the TOWER trial, peripheral blood counts from 267 and 109 patients, respectively, were available at baseline and analyzed in this study. Relapsed or refractory disease was defined as refractory to primary induction therapy or to salvage with intensive combination chemotherapy, first relapse with the first remission less than 12 months, second or greater relapse, or relapse after allogeneic hematopoietic stem cell transplantation (alloHSCT). In the open-label single-arm multicenter phase 2 ALCANTARA trial (NCT02000427), eligible adults (≥ 18 years old) with Ph+ BCP ALL that relapsed or were refractory to at least one second-generation or later tyrosine kinase inhibitor (TKI) (dasatinib, nilotinib, bosutinib, ponatinib) or intolerant to second-generation or later TKIs and intolerant or refractory to imatinib were included. Peripheral blood counts from all the patients (*n* = 45) enrolled in the ALCANTARA trial were available at baseline and analyzed in this study. Eligible patients in both trials had > 5% bone marrow blasts and an Eastern Cooperative Oncology Group (ECOG) performance status ≤ 2. The trial protocols were approved by the ethics committee or institutional review board at each participating center. All the patients provided written informed consent. Complete inclusion and exclusion criteria are published [12,27].

### 2.2. Treatment

In the TOWER trial, eligible patients were randomly assigned 2:1 for treatment with blinatumomab or chemotherapy. The patients received up to two cycles of induction therapy. The responders could receive up to three cycles of consolidation therapy, and if CR, CRh, or CRi was achieved, the patients could receive up to 12 months of maintenance therapy. Induction and consolidation treatments with blinatumomab were administered by continuous intravenous (cIV) infusion for 4 weeks, followed by a 2-week treatment-free interval. For the first week of the first induction cycle, blinatumomab was administered at 9 µg/day, then increased to 28 µg/day for the remaining 3 weeks of cycle 1 and for all 4-week cycles thereafter. Maintenance treatment with blinatumomab was given as a 4-week cIV infusion every 12 weeks. Patients in the blinatumomab arm who had high tumor load during screening received dexamethasone before the start of treatment to prevent cytokine release syndrome. Patients in the chemotherapy arm received one of the following regimens at the investigator’s discretion: fludarabine, high-dose cytosine arabinoside, and granulocyte colony-stimulating factor (G-CSF) with or without anthracycline; a high-dose cytosine arabinoside-based regimen; a high-dose methotrexate-based regimen; or a clofarabine-based regimen. If deemed in the patient’s best interest, a patient could proceed to alloHSCT any time after cycle 1 of induction. In the ALCANTARA trial, eligible patients received up to two cycles of blinatumomab as induction therapy, and if CR, CRh, or CRi was achieved, patients could receive up to three cycles of blinatumomab as consolidation therapy. Blinatumomab was administered with the same dosing regimen as in the TOWER trial. Detailed descriptions on dose modifications, interruptions, and discontinuation are published [12,27].

### 2.3. Assessments

Cytomorphological bone marrow assessments were conducted at the end of each treatment cycle (day 29 of each cycle) to assess hematological response. CR was defined as ≤ 5% bone marrow blasts, no evidence of disease and full recovery of peripheral blood counts (platelets > 100,000/µL and absolute neutrophil count (ANC) > 1000/µL). CRh was defined as CR with partial recovery of peripheral blood counts (platelets > 50,000/µL and ANC > 500/µL). CRi was defined as CR with incomplete recovery of peripheral blood counts (platelets > 100,000/µL or ANC > 1000/µL). Peripheral blood counts were assessed prior to treatment, cycle 1 day 2 (C1 D2), C1 D8, C1 D15, C1 D29, C2 D1, C2 D2, C2 D8, C2 D15, C2 D29, and at the end of treatment (i.e., safety follow-up (SFU) visit 30 days after the last dose of blinatumomab or chemotherapy).

### 2.4. Statistical Analyses

Absolute values of peripheral blood counts were summarized with quartiles at each assessment timepoint for patients who received at least one dose of treatment. The change from baseline to end of treatment in peripheral blood counts was summarized with quartiles and frequencies for patients who achieved CR, CRh, or CRi. White blood cell (WBC), neutrophil, and platelet count changes were reported for the responders (patients achieving CR, CRh, or CRi) and for the entire Ph+ and Ph− patient cohorts (including responders and non-responders).

Analyses of efficacy endpoints included the patients who achieved CR, CRh, or CRi. Event-free survival (EFS) was measured from the time of first CR, CRh, or CRi to hematologic or extramedullary relapse, disease progression, or death from any cause; the patients without an event were censored at their last disease assessment date. OS was measured from the time of the first CR, CRh, or CRi to death resulting from any cause; the patients still alive were censored at the date last known to be alive. EFS and OS were assessed in the responders, comparing the patients achieving CR versus the patients achieving CRh or CRi (this latter group is collectively referred to as “CRh/CRi”). Time-to-event endpoints were summarized with Kaplan–Meier curves and medians. P-values less than 0.05 were considered statistically significant.

## 3. Results

### 3.1. Peripheral Blood WBC, Neutrophil, and Platelet Count Changes with Blinatumomab or Chemotherapy Treatment

Of all the patients enrolled, peripheral blood counts were assessed at baseline, during and after blinatumomab treatment in the patients with Ph− R/R BCP ALL (*n* = 267) and Ph+ R/R BCP ALL (*n* = 45) from the TOWER and ALCANTARA studies, respectively, and during and after chemotherapy in the patients with Ph− R/R BCP ALL from the TOWER study (*n* = 109). The demographic and baseline clinical characteristics of these heavily pretreated patients with R/R BCP ALL are shown in Table 1.

**Figure 1:**
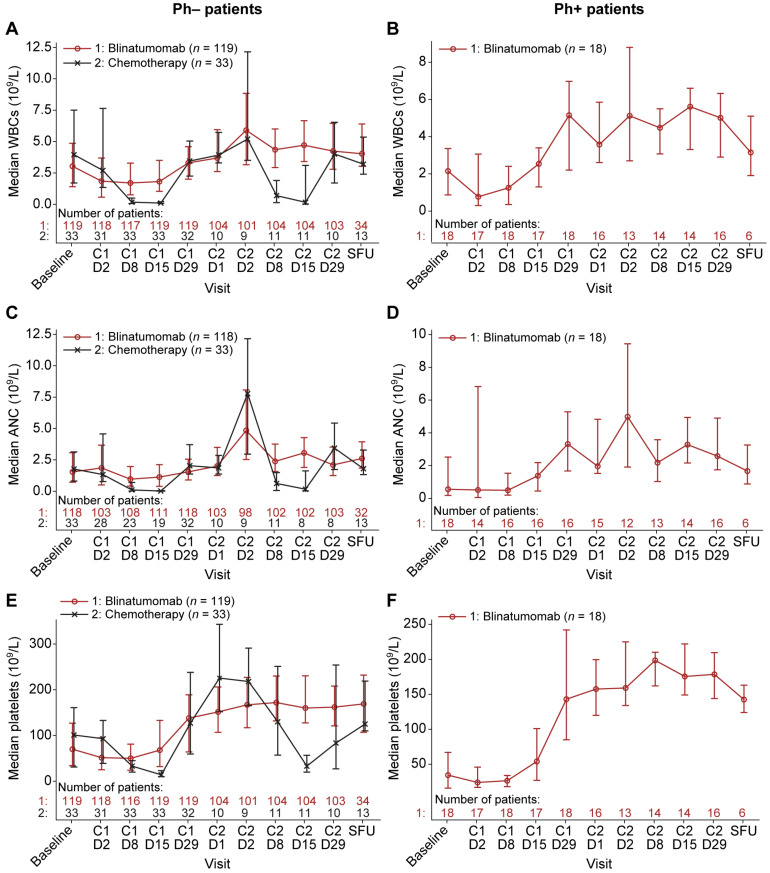
Peripheral blood counts of responders at baseline, during and after two treatment cycles with blinatumomab or chemotherapy. The median WBC of the Ph− (**A**) and Ph+ (**B**) responders, median ANC of the Ph− (**C**) and Ph+ (**D**) responders, and the median platelet count of the Ph− (**E**) and Ph+ (**F**) responders were plotted at baseline, on the cycle and day of treatment assessed, and at the SFU visit 30 days after treatment. Vertical lines represent the first and third quartiles around the median. ANC, absolute neutrophil count; C, cycle; D, day; Ph+, Philadelphia chromosome-positive; Ph−, Philadelphia chromosome-negative; SFU, safety follow-up; WBC, white blood cell.

In the Ph− patients treated with blinatumomab that achieved a response (CR, CRh, or CRi), the median WBC count was 3.03 × 10^9^/L at baseline, dropped 1.8-fold to the nadir of 1.70 × 10^9^/L on day 8 of cycle 1, then gradually increased to 3.30 × 10^9^/L at the end of cycle 1 and 4.24 × 10^9^/L at the end of cycle 2 (Figure 1A).

The median WBC count remained at this level at the SFU visit 30 days after the last dose of blinatumomab. Likewise, in the Ph+ responders, there was a transient drop in the median WBC count during the first cycle of blinatumomab followed by a rapid recovery. The median WBC count dropped 2.8-fold, from 2.15 × 10^9^/L at baseline to the nadir of 0.77 × 10^9^/L at day 2 of cycle 1, reached above baseline levels by day 15 of cycle 1, then increased to 5.01 × 10^9^/L at the end of cycle 2 (Figure 1B). These changes in peripheral blood WBCs in the Ph− (*n* = 119) and Ph+ (*n* = 18) responders during blinatumomab treatment were also seen in the entire Ph− (*n* = 267) and Ph+ (*n* = 45) patient cohorts (responders and non-responders) during blinatumomab treatment (Figure S1). In contrast, in the responders treated with chemotherapy, severe sustained leukopenia occurred following treatment, with recovery not achieved until the end of the treatment cycle. Specifically, in the Ph− responders treated with chemotherapy, the median WBC count markedly dropped 23.2-fold, from 3.95 × 10^9^/L at baseline to 0.17 × 10^9^/L at day 8 and continued to drop to the nadir of 0.10 × 10^9^/L at day 15 of cycle 1 (39.5-fold below baseline), then increased to 3.45 × 10^9^/L at the end of cycle 1. During the second cycle of chemotherapy, the median WBC count markedly dropped 5.7-fold from 3.93 × 10^9^/L at day 1 to 0.69 × 10^9^/L at day 8 and continued to drop to the nadir of 0.16 × 10^9^/L at day 15 (24.6-fold below baseline), then increased to reach 4.01 × 10^9^/L at the end of cycle 2 (Figure 1A). This severe sustained leukopenia following chemotherapy treatment in the Ph− responders (*n* = 33) was also seen in the entire Ph− patient cohort (*n* = 109; Figure S1A). The change in WBC counts from baseline to the end of treatment (i.e., the SFU visit) was also assessed. The median WBC count at the end of treatment exceeded the baseline level by 1.3-fold in the Ph− responders and 1.5-fold in the Ph+ responders treated with blinatumomab but was below baseline in the responders treated with chemotherapy (Figure 1A,B).

In the Ph− responders treated with blinatumomab, the median ANC gradually increased over the two cycles of treatment, from the baseline level of 1.54 × 10^9^/L to 2.10 × 10^9^/L at the end of cycle 2 and to 2.61 × 10^9^/L at SFU (Figure 1C). This gradual increase in the median ANC over two cycles of blinatumomab was likewise observed in the Ph+ responders (Figure 1D) and in the entire Ph− and Ph+ cohorts (Figure S1B). However, in the responders treated with chemotherapy, severe sustained neutropenia occurred following treatment, with recovery not achieved until the end of the treatment cycle. Specifically, in the Ph− responders treated with chemotherapy, the median ANC markedly dropped 16.6-fold from the baseline level of 1.77 × 10^9^/L to 0.11 × 10^9^/L at day 8 and continued to drop to the nadir of 0.02 ×10^9^/L at day 15 of cycle 1 (88.5-fold below baseline), then increased to 2.04 × 10^9^/L by the end of cycle 1 (Figure 1C). This severe sustained neutropenia following chemotherapy treatment in the Ph− responders was also seen for cycle 2 of treatment (Figure 1C) and was observed in the entire Ph− cohort following each of the two cycles of chemotherapy (Figure S1B). At the end of treatment, the median ANC exceeded the baseline level by 1.7-fold in the Ph− responders and 3.0-fold in the Ph+ responders treated with blinatumomab but only reached the baseline level in the responders treated with chemotherapy (Figure 1C,D).

In these heavily pretreated patients with R/R BCP ALL, the median platelet count at baseline was low as the recovery of platelets lags that of other complete blood count parameters following previous chemotherapy. Over the two cycles of treatment of Ph− responders with blinatumomab, the median platelet counts gradually increased from the baseline level of 70 × 10^9^/L to 162 × 10^9^/L at day 29 of cycle 2 (Figure 1E). This platelet count recovery over two treatment cycles of blinatumomab likewise was observed in the Ph+ responders, rising from a median platelet count of 35 × 10^9^/L at baseline to 179 × 10^9^/L at the end of cycle 2 (Figure 1F). However, in the responders treated with chemotherapy, severe sustained thrombocytopenia occurred following treatment, with recovery to near baseline levels at the end of the treatment cycle. In the Ph− responders treated with chemotherapy, the median platelet count decreased from 101 × 10^9^/L at baseline to 33 × 10^9^/L at day 8 and continued to decrease to the nadir of 14 × 10^9^/L at day 15 of cycle 1 (7.2-fold below baseline), rose to 127 × 10^9^/L at the end of cycle 1, decreased again during cycle 2 to the nadir of 33 × 10^9^/L at day 15, then increased by the end of cycle 2, reaching a median of 84 × 10^9^/L (Figure 1E). These trends observed in the responders during treatment with blinatumomab or chemotherapy likewise were observed in the entire Ph− and Ph+ cohorts (Figure S1C). At the end of treatment, the median platelet count exceeded the baseline level by 2.4-fold in the Ph− responders and 4.1-fold in the Ph+ responders treated with blinatumomab but only reached 1.2-fold above baseline in the responders treated with chemotherapy (Figure 1E,F).

These peripheral blood studies demonstrate that treatment of patients with Ph− or Ph+ R/R BCP ALL with two cycles of blinatumomab does not cause persistent leukopenia, neutropenia, or thrombocytopenia. The severe sustained leukopenia, neutropenia, and thrombocytopenia experienced by patients treated with chemotherapy contrasts with the more modest transient leukopenia experienced by patients treated with blinatumomab.

### 3.2. Proportion of CRs among Responders

**Figure 2:**
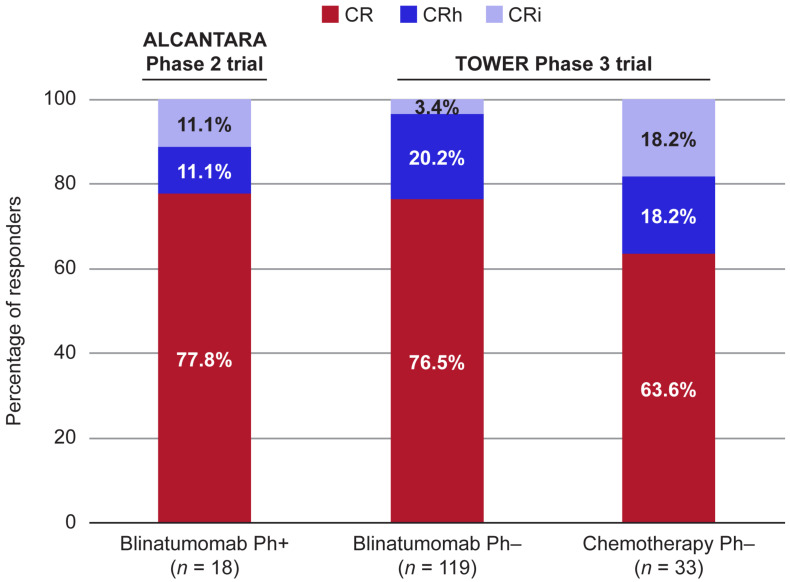
Blinatumomab treatment results in a higher proportion of CR among responders. The proportion of patients achieving CR relative to those achieving CRh/CRi was assessed in the patients with Ph+ R/R BCP ALL treated with blinatumomab from the ALCANTARA trial [27] or the patients with Ph− R/R BCP ALL treated with blinatumomab or chemotherapy from the TOWER trial [12]. ALL, acute lymphoblastic leukemia; BCP, B-cell precursor; CR, complete remission; CRh, CR with partial recovery of peripheral blood counts; CRi, CR with incomplete recovery of peripheral blood counts; Ph+, Philadelphia chromosome-positive; Ph−, Philadelphia chromosome-negative; R/R, relapsed/refractory.

To gauge the degree of bone marrow suppression among the responders, we examined the proportion of patients achieving CR relative to those achieving CRh/CRi. In the Ph+ patients who responded to blinatumomab treatment, 77.8% of the patients achieved CR, and 22.2% of the patients achieved CRh/CRi (11.1% CRh, 11.1% CRi; Figure 2).

Similarly, in the Ph− patients who responded to blinatumomab treatment, 76.5% of the patients achieved CR, and 23.6% of the patients achieved CRh/CRi (20.2% CRh, 3.4% CRi). For the Ph− patients who responded to chemotherapy treatment, 63.6% of the patients achieved CR, and 36.4% of the patients achieved CRh/CRi (18.2% CRh, 18.2% CRi). Although not statistically significant, the proportion of responders achieving CR among the Ph− patients treated with blinatumomab was numerically higher compared with those treated with chemotherapy (76.5% vs. 63.6%; *p* = 0.179).

### 3.3. EFS in Patients Achieving CR versus CRh/CRi

**Figure 3:**
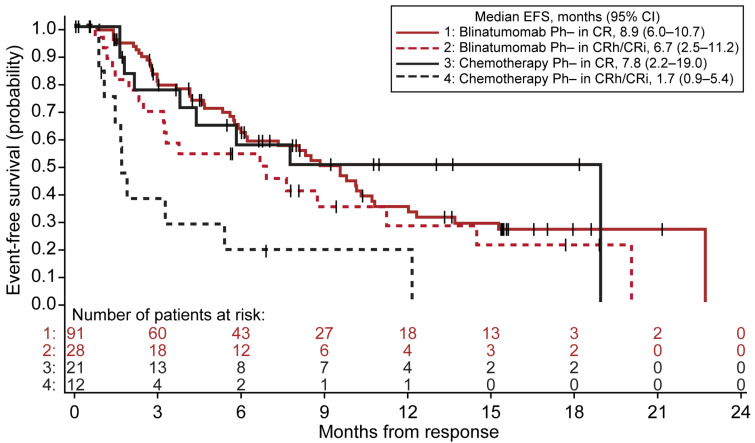
EFS analyzed according to the response within 12 weeks of treatment with blinatumomab or chemotherapy in the patients with Ph− R/R BCP ALL. ALL, acute lymphoblastic leukemia; BCP, B-cell precursor; CI, confidence interval; CR, complete remission; CRh, CR with partial recovery of peripheral blood counts; CRi, CR with incomplete recovery of peripheral blood counts; EFS, event-free survival; Ph−, Philadelphia chromosome-negative; R/R, relapsed/refractory.

In the Ph− patients with R/R BCP ALL treated with blinatumomab or chemotherapy in the TOWER trial, EFS was measured from the time of the first CR, CRh, or CRi to relapse, disease progression, or death from any cause. The duration of EFS was analyzed according to the response, comparing the median EFS in the patients achieving CR versus the patients achieving CRh/CRi. In the Ph− patients treated with blinatumomab, the median EFS from the time of response was similar among the CRh/CRi and CR subgroups (CRh/CRi, 6.7 (95% CI, 2.5–11.2) months vs. CR, 8.9 (95% CI, 6.0–10.7) months; *p* = 0.146; Figure 3, Table S1).

However, in the patients who responded to chemotherapy treatment, the median EFS was significantly shorter in the CRh/CRi subgroup compared with the CR subgroup (CRh/CRi, 1.7 (95% CI, 0.9–5.4) months vs. CR, 7.8 (95% CI, 2.2–19.0) months; *p* = 0.004; Figure 3, Table S2).

### 3.4. OS in Patients Achieving CR versus CRh/CRi

**Figure 4:**
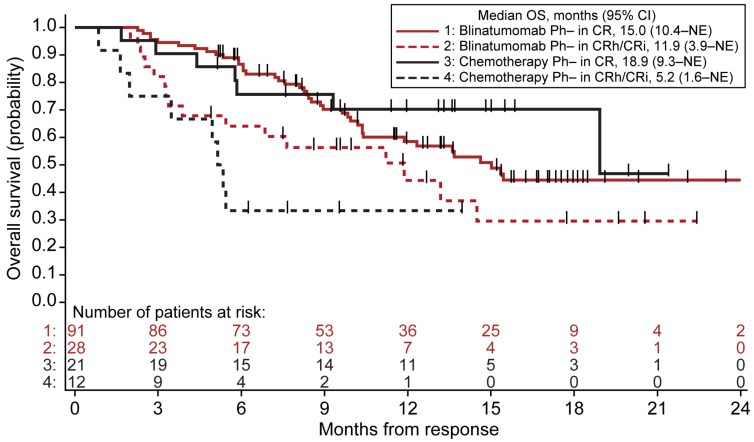
OS analyzed according to the response within 12 weeks of treatment with blinatumomab or chemotherapy in the patients with Ph− R/R BCP ALL. ALL, acute lymphoblastic leukemia; BCP, B-cell precursor; CI, confidence interval; CR, complete remission; CRh, CR with partial recovery of peripheral blood counts; CRi, CR with incomplete recovery of peripheral blood counts; NE, not estimable; OS, overall survival; Ph−, Philadelphia chromosome-negative; R/R, relapsed/refractory.

OS was measured from the first dose of treatment to death resulting from any cause for the Ph− patients with R/R BCP ALL treated with blinatumomab or chemotherapy in the TOWER trial. The duration of OS was analyzed according to the response, comparing the median OS in the patients achieving CR versus the patients achieving CRh/CRi. The patients treated with blinatumomab who achieved CRh/CRi had a numerically lower median OS from the time of response, but not significant, than the patients achieving CR (CRh/CRi, 11.9 (95% CI, 3.9–NE) months vs. CR, 15.0 (95% CI, 10.4–NE) months; *p* = 0.062; Figure 4, Table S3).

The patients treated with chemotherapy who achieved CRh/CRi had a significantly lower median OS versus the patients achieving CR (CRh/CRi, 5.2 (95% CI, 1.6–NE) months vs. CR, 18.9 (95% CI, 9.3–NE) months; *p* = 0.013; Figure 4, Table S4).

## 4. Discussion

Myelosuppression is characterized by the suppression of normal bone marrow function that leads to a decrease in production of blood cells. Chemotherapy-induced myelosuppression is a potentially fatal complication of cancer therapy and is caused by the destruction of hematopoietic progenitor cells that produce mature blood cells, including WBCs and platelets. Upon treatment with high doses of cytotoxic chemotherapy and radiation therapy, immature bone marrow cells as well as the existing mature cells are eliminated, resulting in myelosuppression [28]. With blinatumomab, although a rapid and transient decrease in lymphocytes was observed within the first few hours of treatment, this was followed by an accelerated increase in T cells [20,29]. Normal hematopoietic progenitor cells upstream of B-lymphoid progenitor cells do not express CD19; hence, blinatumomab does not have any effect on these cells [30,31]. This unique aspect of blinatumomab’s mechanism of action allows for the recovery of normal hematopoiesis, thereby avoiding myelosuppression, while eliminating CD19-expressing leukemia cells.

The key findings of this study indicated that treatment with blinatumomab did not cause persistent myelosuppression in heavily pretreated adult patients with R/R Ph− BCP ALL (*n* = 267) and R/R Ph+ BCP ALL (*n* = 45). The median WBC, ANC, and platelet count gradually increased over two cycles of treatment with blinatumomab in all the patients and in the responders, with the median WBC and ANC reaching above baseline at the end of cycle 2. However, in the patients treated with cytotoxic chemotherapy, the median WBC, ANC, and platelet count decreased at the beginning of each treatment cycle, remained at the nadir until the end of the second week of treatment, and then increased to the near baseline level at the end of treatment. The difference in the incidence of myelosuppression in patients treated with blinatumomab and chemotherapy could be postulated to be even higher considering that 45% of the Ph− patients in the chemotherapy arm of the TOWER study were treated with a chemotherapy regimen consisting of G-CSF along with fludarabine and high-dose cytosine arabinoside with or without anthracycline [12]. These findings demonstrate that blinatumomab is minimally and transiently myelosuppressive, which allows patients to recover from bone marrow suppression due to prior chemotherapy, thereby leading to lower rates of serious and potentially life-threatening infections. In the TOWER study, 34.1% and 52.3% of patients treated with blinatumomab and chemotherapy, respectively, experienced grade ≥ 3 infections that were reported in at least 3% of the patients [12]. In addition, results from a phase 3 multicenter randomized trial conducted by the Children’s Oncology Group demonstrated that blinatumomab was associated with a lower rate of febrile neutropenia and sepsis compared with chemotherapy (5% vs. 58% and 2% vs. 27%, respectively) [13]. In contrast, cytotoxic chemotherapy including inotuzumab ozogamicin, a cytotoxic CD22-targeting antibody drug conjugate used for treatment of R/R BCP ALL, has been shown to be myelosuppressive [32,33]. Adult patients with R/R BCP ALL treated with chemotherapy or inotuzumab ozogamicin exhibited high rates of febrile neutropenia (chemotherapy, 49%; inotuzumab ozogamicin, 24%), thrombocytopenia (chemotherapy, 59%; inotuzumab ozogamicin, 37%), and leukopenia (chemotherapy, 39%; inotuzumab ozogamicin, 25%) [32].

Additionally, non-myelosuppressive therapies may be critically important for the treatment of cancer patients during the COVID-19 pandemic as the mortality rate due to COVID-19 among cancer patients is reported to be > 30% [17]. Since T cells are important for anti-SARS-CoV-2 immunity [34,35,36,37] and blinatumomab does not suppress T-cell function, patients treated with blinatumomab instead of chemotherapy might be expected to have a lower risk of infection by SARS-CoV-2 and death due to COVID-19. B-cell function also plays an important role in anti-SARS-CoV-2 immunity [37,38,39]. Hypogammaglobulinemia, a well-known side effect of cytotoxic chemotherapy, blinatumomab treatment, and anti-CD19 CAR-T cell therapy, is a risk factor for severe and prolonged COVID-19 [40,41]. However, it is noteworthy that B-cell depletion and hypogammaglobulinemia due to blinatumomab treatment are transient and reversible [42], which may be more advantageous when compared to anti-CD19 CAR-T therapies that can cause profound and prolonged B-cell aplasia [43,44].

In comparison to patients treated with chemotherapy, there is a trend towards a higher proportion of responding patients treated with blinatumomab achieving CR than those achieving CRh/CRi in this study. Furthermore, the patients who achieved CRh/CRi versus CR after treatment with blinatumomab had similar efficacy outcomes, including median EFS (6.7 vs. 8.9 months, respectively) and OS (11.9 vs. 15.0 months, respectively). In contrast, the patients who achieved CRh/CRi upon treatment with chemotherapy had poorer efficacy outcomes than those who achieved CR, including significantly shorter median EFS (1.7 vs. 7.8 months) and median OS (5.2 vs. 18.9 months). The latter finding is consistent with results from other studies in adult patients with ALL treated with cytotoxic chemotherapy or cytotoxic inotuzumab ozogamicin that demonstrate that CRh/CRi was associated with worse OS when compared with CR [15,32]. Collectively, these findings indicate that while CRh/CRi is associated with poorer outcomes in comparison to CR for cytotoxic chemotherapy and inotuzumab ozogamicin treatment, CRh/CRi could prove to be a clinically meaningful endpoint for blinatumomab treatment that predicts outcomes similar to CR.

The study had a few limitations. Firstly, there was no comparator arm in the ALCANTARA study. Secondly, although there was a trend that a greater proportion of Ph− patients achieved CR with blinatumomab versus chemotherapy, the difference was not statistically significant. Additional data are needed to further validate the observation that CRh/CRi could be used as a meaningful efficacy endpoint similar to CR in patients treated with blinatumomab.

## 5. Conclusions

In conclusion, our studies show that blinatumomab treatment causes only modest and transient myelosuppression, unlike chemotherapy. Furthermore, the patients who achieved CRh/CRi upon treatment with blinatumomab attained similar EFS and OS benefits to those who achieved CR, suggesting that CRh/CRi may be a clinically meaningful endpoint similar to CR in blinatumomab treatment.

## Figures and Tables

**Table 1:** Demographic and baseline clinical characteristics of patients in the phase 3 TOWER and phase 2 ALCANTARA clinical trials.

Characteristic	TOWER Phase 3 Trial [12]		ALCANTARA Phase 2 Trial [27]
	Blinatumomab Arm (*n* = 271)	Chemotherapy Arm (*n* = 134)	Blinatumomab Arm (*n* = 45)
Age—years			
Mean (range)	40.8 (18–80)	41.1 (18–78)	52.8 (23–78)
Sex—*n* (%)			
Male	162 (59.8)	77 (57.5)	24 (53.3)
Female	109 (40.2)	57 (42.5)	21 (46.7)
ECOG performance status—*n* (%)			
0	96 (35.4)	52 (38.8)	16 (35.6)
1	134 (49.4)	61 (45.5)	20 (44.4)
2	41 (15.1)	20 (14.9)	9 (20.0)
Missing data	0	1 (0.7)	N/A
Prior alloHSCT—*n* (%)			
Yes	94 (34.7)	46 (34.3)	20 (44.4)
No	176 (64.9)	87 (64.9)	25 (55.6)
Unknown	1 (0.4)	1 (0.7)	N/A
Prior salvage regimen—*n* (%)			
0	N/A	N/A	14 (31.1)
1	114 (42.1)	65 (48.5)	12 (26.7)
2	91 (33.6)	43 (32.1)	11 (24.4)
≥3	66 (24.4)	26 (19.4)	8 (17.8)
Prior tyrosine kinase inhibitor treatment—*n* (%)			
1	N/A	N/A	7 (15.6)
2	N/A	N/A	21 (46.7)
3	N/A	N/A	13 (28.9)
4	N/A	N/A	4 (8.9)
Bone marrow blasts—*n* (%)			
<10%	9 (3.3)	7 (5.2)	2 (4.4)
10 to <50%	60 (22.1)	23 (17.2)	9 (20.0)
≥50%	201 (74.2)	104 (77.6)	34 (75.6)
Unknown	1 (0.4)	0	N/A

Note: alloHSCT, allogeneic hematopoietic stem cell transplantation; ECOG, Eastern Cooperative Oncology Group; N/A, not applicable.

## Data Availability

Qualified researchers may request data from Amgen clinical studies. Complete details are available at http://www.amgen.com/datasharing (accessed on 6 November 2021). Please contact Amgen Inc. for additional details.

## Supplementary Materials

The following are available online at https://www.mdpi.com/article/10.3390/cancers13225607/s1, Figure S1: Peripheral blood counts of patients with R/R BCP ALL at baseline and during two treatment cycles with blinatumomab or chemotherapy, Table S1: EFS analyzed according to response within 12 weeks of treatment with blinatumomab, Table S2: EFS analyzed according to response within 12 weeks of treatment with chemotherapy, Table S3: OS analyzed according to response within 12 weeks of treatment with blinatumomab, Table S4: OS analyzed according to response within 12 weeks of treatment with chemotherapy.
