# Supplementary material for: Impact of Blinatumomab Treatment on Bone Marrow Function in Patients with Relapsed/Refractory B-Cell Precursor Acute Lymphoblastic Leukemia
Source: Cancers (Basel). 2021 Nov 9;13(22):5607. doi: 10.3390/cancers13225607 (PMC8616108; doi:10.3390/cancers13225607)
Supplement: Supplementary file 1 [file cancers-13-05607-s001.zip › cancers-1417229-supplementary.pdf]

Supplementary materials:

Impact of Blinatumomab Treatment on Bone Marrow Function in Patients with Relapsed/Refractory B Cell Precursor Acute Lymphoblastic Leukemia

Hagop M. Kantarjian [1], Gerhard Zugmaier [2,*,†], Monika Brüggemann [3], Brent L. Wood [4], Heinz A. Horst [5], Yi Zeng [6] and Giovanni Martinelli [7]

A. Median WBC

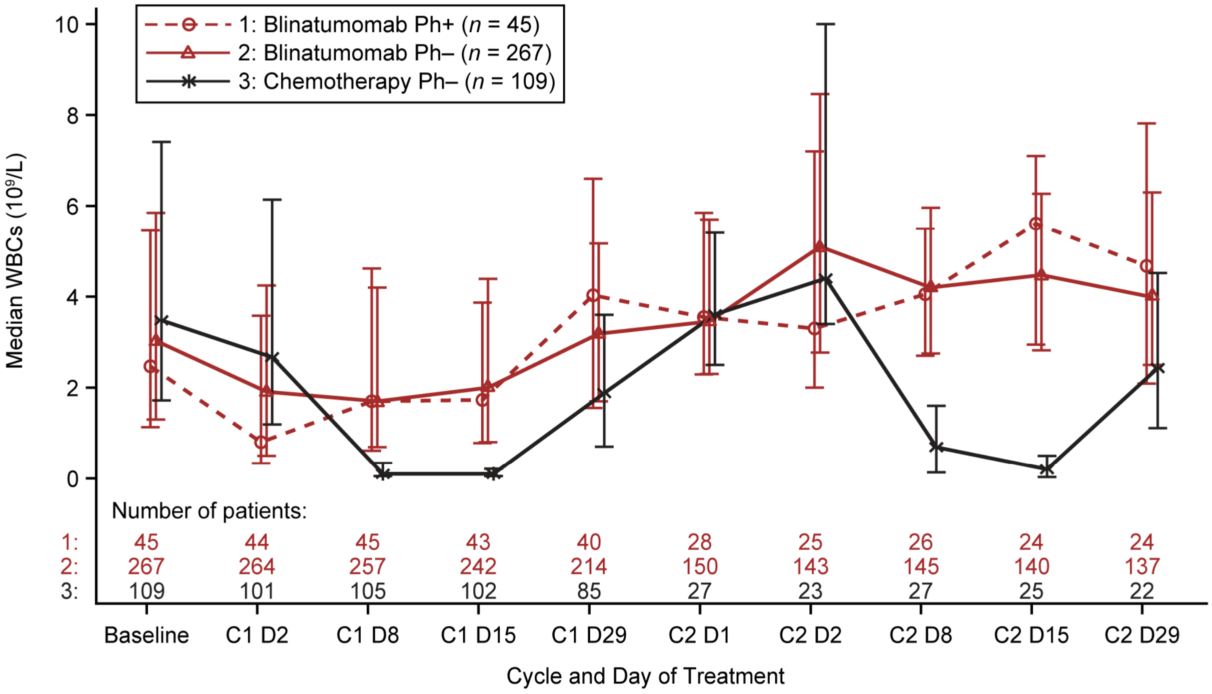

B. Median ANC

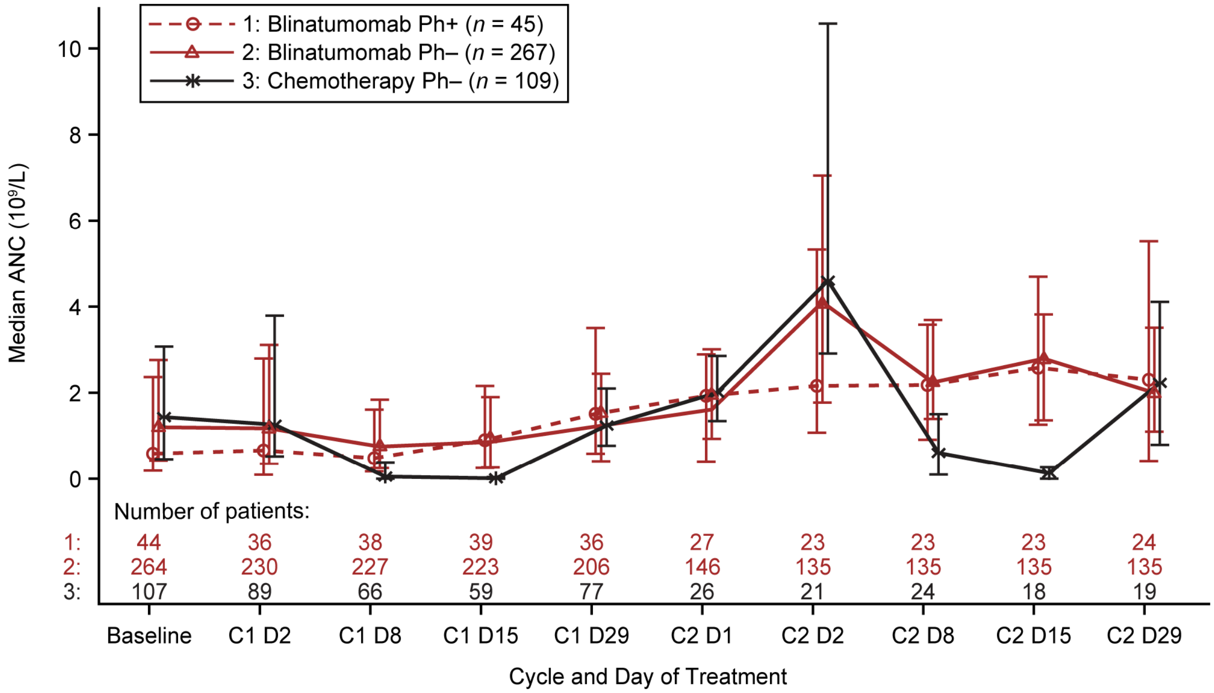

C. Median platelet count

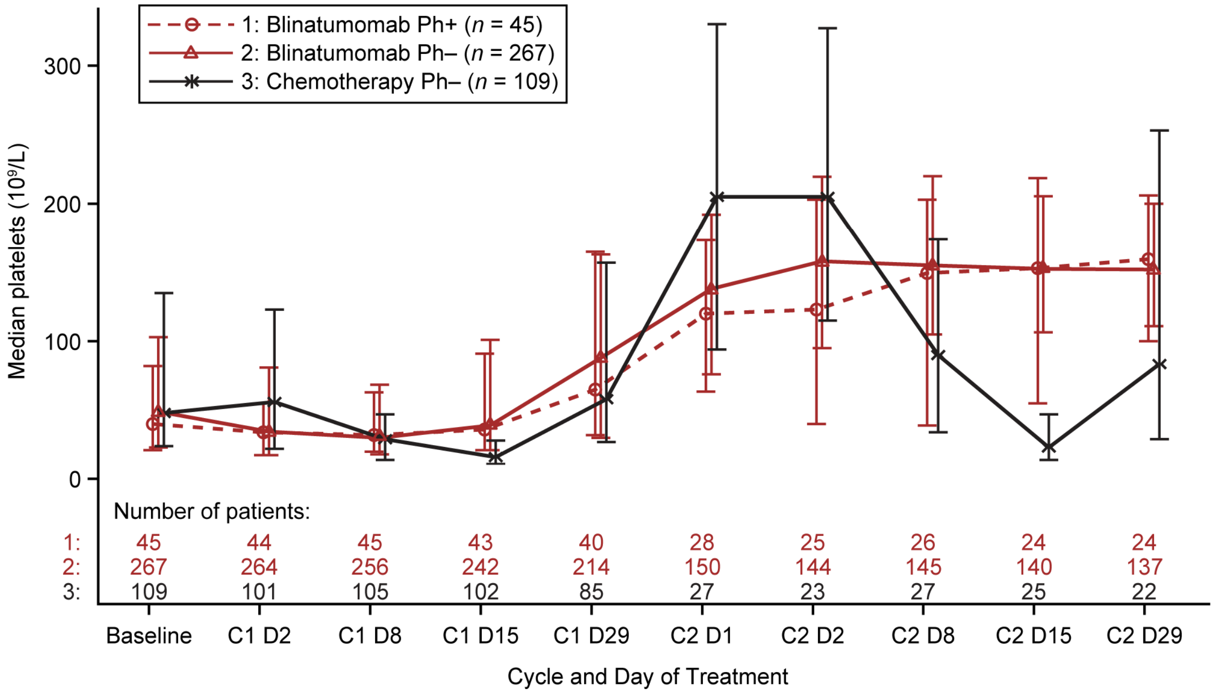

Figure S1. Peripheral blood counts of patients with R/R BCP ALL at baseline and during two treatment cycles with blinatumomab or chemotherapy. Peripheral blood counts were assessed at baseline, during and after treatment of all patients with R/R BCP ALL with blinatumomab (*N* = 312; *n* = 267 Ph- patients from the TOWER trial [12], *n* = 45 Ph+ patients from the ALCANTARA trial [27]) or chemotherapy (*N* = 109 from the TOWER trial). The median WBC (**A**), ANC (**B**), and platelet count (**C**) were plotted at baseline and on the cycle and day of treatment assessed. Vertical lines represent the first and third quartiles around the median. ALL, acute lymphoblastic leukemia; ANC, absolute neutrophil count; BCP, B-cell

precursor; C, cycle; D, day; Ph+, Philadelphia chromosome–positive; Ph-, Philadelphia chromosome–negative; R/R, relapsed/refractory; WBC, white blood cell.

Table S1. EFS analyzed according to response within 12 weeks of treatment with blinatumomab.

**Heading**	**Achieved CR (*N* = 91)**	**Achieved CRh/CRi (*N* = 28)**
Patient status		
Number of patients	91	28
Events – *n* (%)	49 (53.8)	19 (67.9)
Relapse	27 (29.7)	6 (21.4)
Progressive disease	4 (4.4)	3 (10.7)
Extramedullary relapse	0 (0.0)	2 (7.1)
Death from any cause	18 (19.8)	8 (28.6)
Time-to-event (KM) (months)[a]		
Median	8.9	6.7
95% CI (median)	(6.0–10.7)	(2.5–11.2)
Q1, Q3	4.2, 22.7	2.3, 14.5
Min, max	0.8, 22.7	0.8, 20.1

CI, confidence interval; CR, complete remission; CRh, CR with partial recovery of peripheral blood counts; CRi, CR with incomplete recovery of peripheral blood counts; EFS, event-free survival; KM, Kaplan-Meier; Q, quartile. [a] Months are calculated as days from first response date to event/censor date, divided by 30.5.

Table S2. EFS analyzed according to response within 12 weeks of treatment with chemotherapy.

Heading	Achieved CR (*N* = 21)	Achieved CRh/CRi (*N* = 12)
Patient status		
Number of patients	21	12
Events – n (%)	9 (42.9)	10 (83.3)
Relapse	5 (23.8)	3 (25.0)
Progressive disease	0 (0.0)	1 (8.3)
Extramedullary relapse	0 (0.0)	1 (8.3)
Death from any cause	4 (19.0)	5 (41.7)
Time-to-event (KM) (months)[a]		
Median	7.8	1.7
95% CI (median)	(2.2–19.0)	(0.9–5.4)
Q1, Q3	3.8, 19.0	1.1, 5.4
Min, max	1.6, 19.0	0.9, 12.2

CI, confidence interval; CR, complete remission; CRh, CR with partial recovery of peripheral blood counts; CRi, CR with incomplete recovery of peripheral blood counts; EFS, event-free survival; KM, Kaplan-Meier; Q, quartile.
[a] Months are calculated as days from first response date to event/censor date, divided by 30.5.

Table S3. OS analyzed according to response within 12 weeks of treatment with blinatumomab.

Heading	Achieved CR (*N* = 91)	Achieved CRh/CRi (*N* = 28)
Patient status		
Number of patients	91	28
Events – n (%)	40 (44.0)	16 (57.1)
Death from any cause	40 (44.0)	16 (57.1)
Time-to-event (KM) (months)[a]		
Median	15.0	11.9
95% CI (median)	(10.4–NE)	(3.9–NE)
Q1, Q3	8.4, NE	3.3, NE
Min, max	2.3, 15.4	2.0, 14.5

CI, confidence interval; CR, complete remission; CRh, CR with partial recovery of peripheral blood counts; CRi, CR with incomplete recovery of peripheral blood counts; KM, Kaplan-Meier; NE, not estimable; OS, overall survival; Q, quartile. [a] Months are calculated as days from first response date to event/censor date, divided by 30.5.

Table S4. OS analyzed according to response within 12 weeks of treatment with chemotherapy.

Heading	Achieved CR (*N* = 21)	Achieved CRh/CRi (*N* = 12)
Patient status		
Number of patients	21	12
Events – n (%)	7 (33.3)	8 (66.7)
Death from any cause	7 (33.3)	8 (66.7)
Time-to-event (KM) (months)[a]		
Median	18.9	5.2
95% CI (median)	(9.3–NE)	(1.6–NE)
Q1, Q3	9.3, NE	2.7, NE
Min, max	1.7, 18.9	0.9, 5.4

CI, confidence interval; CR, complete remission; CRh, CR with partial recovery of peripheral blood counts; CRi, CR with incomplete recovery of peripheral blood counts; KM, Kaplan-Meier; NE, not estimable; OS, overall survival; Q, quartile. [a] Months are calculated as days from first response date to event/censor date, divided by 30.5.
